# Bridging the Gaussian extremes: the economic and regulatory imperative of synthetic data in cardiovascular imaging

**DOI:** 10.1093/ehjimp/qyag053

**Published:** 2026-04-30

**Authors:** Carlo Alberto Carnevale Maffè

**Affiliations:** SDA Bocconi School of Management, Milan, Italy

## Navigating the dual extremes of the cardiovascular imaging Gaussian

The contributions to this journal have been building a methodological bridge between two seemingly disparate ends of the cardiovascular imaging spectrum: the high-intensity technical engineering of acquisition physics and the granular, high-stakes environment of clinical decision-making. The ‘Gaussian distribution’ of imaging—stretching from the mathematics of pulse sequences to the bedside management of complex heart failure—requires a unifying medium that can scale across both domains. From an economic perspective, one must observe that the most promising medium emerging today is the utilization of synthetic data.

Synthetic data refer to information that is algorithmically generated to mimic the statistical properties, patterns, and features of real-world data, but which does not correspond to any specific individual. In the context of cardiovascular imaging, this means creating ‘virtual patients’ whose cardiac magnetic resonance (CMR), computed tomography (CT), or echocardiographic scans are indistinguishable from real clinical cases to an artificial intelligence (AI) model, yet carry none of the privacy or legal baggage associated with sensitive health information. This technological leap is not merely a technical curiosity; it is a fundamental economic necessity for the sustainability of European healthcare systems.

## The productivity crisis in cardiology

To understand why synthetic data is economically transformative, one must first address a persistent ailment in the service sector known as Baumol’s cost disease. Identified in the 1960s, this economic principle explains why costs in labour-intensive sectors like healthcare rise faster than general inflation despite seemingly stagnant productivity. In manufacturing, a technician can produce more units per hour as machinery improves, allowing wages to rise while unit costs fall. In cardiology, however, the ‘service’ of diagnosing a patient is often the labour itself. A cardiologist in 2026 takes roughly the same amount of time to perform a high-quality interpretation of a complex echocardiogram as they did a decade ago. From a quantitative economic perspective, the ‘cost explosion’ described by Baumol is evident in current European fiscal data. In 2023, healthcare expenditure in the EU reached €1.72 trillion, or 10.0% of the Union's GDP. Within this landscape, cardiovascular diseases alone impose an annual burden of €282 billion, equivalent to roughly €630 per capita, with health and long-term care accounting for 55% of these costs. This financial pressure is intensified by a persistent productivity gap; whereas average labour productivity across the OECD grew by only 0.6% in 2023, the healthcare sector—where curative and rehabilitative services account for 52.7% of spending—remains fundamentally labour-dependent. Without AI-driven industrialization, the necessity to provide competitive wages to a workforce already facing a shortage of 1.2 million professionals will continue to drive unit costs upward by an estimated 1% to 5% annually.

The integration of AI, fuelled by synthetic data, offers the first true ‘cure’ for Baumol’s disease in medicine. By transforming the labour-intensive diagnostic service into a digital ‘good’ that can be industrialized, healthcare systems can finally decouple productivity from human hours. Synthetic data accelerate this by providing the massive datasets required to train AI systems that can automate routine tasks—such as semantic segmentation of myocardial images or stenosis quantification—allowing clinicians to focus on the ‘long tail’ of complex cases where human judgment remains paramount.

## The technical end of the Gaussian: the mathematics of synthesis

The mechanism of synthetic data generation is a study in computational elegance. Historically, medical imaging research relied on data augmentation—simple techniques like rotating or scaling existing images to increase dataset size. Modern synthesis, however, employs deep generative models that learn the underlying ‘manifold’ or mathematical structure of cardiac anatomy and pathology. The evolution from generative adversarial networks (GANs) to denoising diffusion probabilistic models represent a significant shift in the technical end of our Gaussian. A GAN utilizes two networks—a generator and a discriminator—locked in a competitive game where the generator tries to create realistic images to ‘fool’ the discriminator. While effective, GANs can suffer from ‘mode collapse’, where the variety of generated images is limited. Conversely, diffusion models—the technology behind state-of-the-art generators like NVIDIA’s MAISI—work through a reverse-diffusion process. They take pure Gaussian noise and, through a series of learned steps, ‘denoise’ it into a high-fidelity medical image. For cardiovascular imaging, this allows for the generation of 3D CT volumes with precise ‘voxel’ (volume pixel) resolution (e.g. 512 × 512 × 512) that accurately represent rare anomalies, such as specific coronary artery variants or complex congenital malformations, which are statistically underrepresented in real-world databases.

## The clinical end of the Gaussian: time savings and workflow efficiency

At the clinical end of the Gaussian, the primary metric of success is the ‘Time to Answer’. In acute cardiovascular care, every minute saved in the diagnostic pathway translates directly into better patient outcomes and lower systemic costs. AI and synthetic data contribute to these time savings through three primary channels: acquisition guidance, automated post-processing, and administrative streamlining. In echocardiography, deep learning algorithms trained on synthetic variations of probe angles can provide real-time guidance to inexperienced operators (such as nurses in resource-limited settings). This ensures that ‘first-time-right’ images are captured, reducing the need for repeat scans and minimizing patient discomfort. For complex modalities like CMR, AI can automate scan planning and shimming (the process of homogenizing the magnetic field), reducing the examination time by several minutes. The most significant efficiency gains, however, occur in the post-processing phase. The manual segmentation of the left ventricle to calculate ejection fraction or mass is a tedious task that can take up to 30 min per patient. AI-driven tools, validated on synthetic datasets that represent a diverse range of heart shapes and sizes, can perform this task in seconds with accuracy comparable to or exceeding that of board-certified cardiologists.

## Economic sustainability: scaling innovation in the EU

From a macroeconomic perspective, the European healthcare sector is facing a ‘scissors crisis’: the demand for imaging is rising due to an ageing population, while the supply of skilled radiologists and cardiologists is constrained. Synthetic data allows European medical device manufacturers and startups to ‘scale up’ their AI solutions without the prohibitively high costs of acquiring and labelling real-world data. Projects such as the European initiative ‘Project SEARCH’ exemplify this strategy. By creating a secure ecosystem for the generation of high-quality synthetic datasets for cardiovascular and oncological diseases, SEARCH aims to bypass traditional data-sharing barriers like institutional silos and privacy concerns. This ‘democratization’ of data access is essential for a competitive European economy, ensuring that smaller centres and startups can innovate at the same pace as large multinational corporations. Furthermore, synthetic data address the economic cost of ‘rare disease bias’. Traditional AI models trained on majority populations often fail when presented with rare cardiovascular conditions. By using synthetic data to ‘balance’ training sets, we can create AI diagnostic tools that are more robust and equitable, reducing the long-term costs associated with misdiagnosis or late-stage treatment in underrepresented groups.

## Legal compliance and the regulatory landscape

The utilization of synthetic data is not only an economic boon but also a strategic response to the complex European regulatory environment. The General Data Protection Regulation (GDPR) and the newly enacted EU AI Act create a high bar for the use of personal health data in research and clinical AI development. Fully synthetic data—generated entirely from scratch and devoid of any identifiers—is generally considered to fall outside the scope of the GDPR because it does not pertain to an identifiable natural person. This allows for the ‘secondary use’ of data—repurposing insights for research and innovation—without the need for the arduous and often impossible task of obtaining retrospective consent from thousands of patients. Importantly, the EU AI Act classifies most cardiovascular diagnostic AI as ‘high-risk’. Article 10 of the AI Act mandates that high-risk systems be trained on high-quality, representative, and unbiased datasets. Paragraph 5 of Article 10 specifically allows for the processing of sensitive data categories to detect and correct algorithmic bias. Synthetic data is explicitly recognized as a viable solution for fulfilling these requirements, acting as a privacy-preserving mechanism to ensure that AI models are both technically robust and socially fair.

## Addressing the risks: trust and domain shift

As we navigate the synthetic frontier, we must remain cognizant of the limitations that exist at both ends of our Gaussian. For the clinician, the primary concern is trust: ‘Can I rely on an algorithm trained on ‘fake’ data?’. For the technician, the concern is ‘domain shift’—the statistical discrepancy between synthetic training data and real-world clinical images. The solution lies in rigorous validation. Models like NVIDIA’s MAISI utilize Fréchet Inception Distance (FID) scores to mathematically quantify how close synthetic images are to the real distribution. A lower FID score indicates higher fidelity. Moreover, hybrid approaches—mixing real and synthetic data—have been shown to improve the ‘generalization’ of AI models, making them more resilient when applied to new, unseen patient populations from different hospital systems. Furthermore, we must avoid ‘memorization,’ where a generative model accidentally replicates a real patient's unique features. Technical safeguards, such as membership inference attacks and nearest-neighbour probes, are essential components of the ‘technical end’ to ensure that synthetic data remains truly anonymous.

## Environmental sustainability: the ‘green AI’ dimension

The economic sustainability of cardiovascular imaging also encompasses its environmental footprint. The high computational demand of training state-of-the-art Diffusion models contributes to energy consumption and electronic waste. However, AI can also act as a powerful tool for environmental optimization. By reducing the need for repeat scans through AI-guided ‘first-time-right’ acquisition, we lower the total energy expenditure per patient journey. Moreover, AI-enabled telemedicine and remote monitoring—supported by synthetic-data-driven algorithms—can significantly lower the need for physical infrastructure use and patient travel, aligning healthcare delivery with broader European environmental goals (*Table 1*).

**Table 1:** Estimated time savings and efficiency gains via AI and synthetic data

Imaging modality	Manual/traditional time	AI-augmented time	Efficiency gain (%)	Clinical benefit
Echo acquisition	15–20 min	10–12 min	33%−40%	Operator independence
CMR segmentation	20–40 min	<1 min	>95%	Quantitative precision
CCTA interpretation	30 min	5 min	83%	Faster triage in ER
4D flow MRI analysis	Hours (Research)	Minutes	Significant	Haemodynamic insight
Summary of records	7 min	16 s	>96%	Reduced administrative load

Synthetic data should not be seen as a replacement for clinical expertise but as the ‘industrial revolution’ of the cardiovascular imaging sector. It addresses the productivity stagnation of Baumol’s cost disease, provides a legal sanctuary within the strictures of the GDPR and AI Act, and democratizes the ability to innovate across the European Health Data Space. Whether a professional occupies the technical or the clinical end of the imaging Gaussian, synthetic data are the unifying thread. It allows the physicist to test the limits of what can be acquired and the clinician to test the limits of what can be diagnosed, all while protecting the most sacred element of the medical profession: the privacy and trust of patients. The next decade of cardiovascular imaging will not be defined by the number of scans we perform, but by the intelligence with which we synthesize and apply the vast ocean of data at our disposal. This is the true path to a sustainable, equitable, and high-performing European healthcare system. In this new era, the ‘Methods’ will increasingly include the mathematics of synthesis, and the ‘Practice’ will be defined by the seamless integration of these digital twins into the daily life of the cardiology clinic. By bridging these extremes, we are not just imaging the heart; we are reimagining the very economics of care.

## Data Availability

Data available on request.

